# COVID-19 Vaccine Hesitancy in a City with Free Choice and Sufficient Doses

**DOI:** 10.3390/vaccines9111250

**Published:** 2021-10-28

**Authors:** Martin C. S. Wong, Eliza L. Y. Wong, Annie W. L. Cheung, Junjie Huang, Christopher K. C. Lai, Eng Kiong Yeoh, Paul K. S. Chan

**Affiliations:** 1 JC School of Public Health, Faculty of Medicine, The Chinese University of Hong Kong, Hong Kong; wong_martin@cuhk.edu.hk (M.C.S.W.); junjie_huang@link.cuhk.edu.hk (J.H.); 2 Centre for Health Systems & Policy Research, JC School of Public Health, Faculty of Medicine, The Chinese University of Hong Kong, Hong Kong; lywong@cuhk.edu.hk (E.L.Y.W.); anniewlcheung@cuhk.edu.hk (A.W.L.C.); 3 Department of Microbiology, Faculty of Medicine, The Chinese University of Hong Kong, Hong Kong; chris.kclai@cuhk.edu.hk

**Keywords:** COVID-19, vaccine hesitancy, vaccine choice, barriers, incentives, compulsory vaccination

## Abstract

Background: Vaccine hesitancy represents one of the major global health issues around the world. We examined the perception, attitude, perceived barriers and facilitation measures of receiving the COVID-19 vaccine in a Chinese population with free vaccine choices (Sinovac [Coronavac] vs. BioNTech/Fosun [Comirnaty]) and adequate doses. Method: We conducted a random telephone survey of the general population in 1195 subjects aged 18 years or above from 23 April 2021 to 8 May 2021 after two months of vaccine rollout. A descriptive analysis of the levels of enabling factors, obstacles and perception of COVID-19 vaccination was conducted using ANOVA and Chi-square tests for trend. Results: Only 10.1% and 13.5% had received one and two COVID-19 vaccine doses, respectively. Among those who had not received any COVID-19 vaccine (75.4%), only 25.1% expressed their intention to receive in the coming 6 months. The barriers with the highest scores included “having heard of cases with serious adverse events or death after vaccination” (score: 8.17 out 10, 95% C.I. 7.99, 8.35), “lack of confidence on governmental recommendations” (7.69, 95% C.I. 7.47, 7.91), and “waiting for a better vaccine” (7.29, 95% C.I. 7.07, 7.52). The highest score for the impact of various incentives for vaccination was for “vaccine passports for overseas travel” (4.44, 95% C.I. 4.18, 4.71). Conclusions: Vaccine hesitancy is commonly observed in this Chinese population despite adequate provision of vaccine doses and choices. No single incentive is strong enough to promote vaccination, and multiple facilitation measures for different groups of population are needed to encourage vaccine uptake. Active clarification and promotion by medical professionals together with a variety of incentives are needed to drive vaccine uptake.

## 1. Introduction

The coronavirus disease 2019 (COVID-19) has spread globally and became a pandemic of public concern since it was first recognized in December 2019 [1,2]. After one and a half year of pandemic, as of June 2021; it has affected more than 179 million cases and caused greater than 3.9 million deaths worldwide [3]. COVID-19 has induced a heavy burden on health and economy globally, and the availability of COVID-19 vaccines was perceived as the best hope for mitigation of the pandemic. As of June 2021, more than 196 countries have started vaccination against COVID-19. Approximately 2.8 billion doses of COVID-19 have been administered, and 40.5 million are now administered each day globally. There are 22.2% of the world population that has received at least one dose of the COVID-19 vaccine [4]. While much progress has been made in COVID-19 vaccine production, vaccine hesitancy could be another obstacle in achieving a high coverage.

Prior to the emergence of COVID-19, the World Health Organization (WHO) proposed vaccine hesitancy as one of the 10 major global threats to health in 2019 [5]. The WHO defined vaccine hesitancy as a “delay in acceptance or refusal of safe vaccines despite availability of vaccine services” [6]. Vaccine hesitancy is related to different factors that vary by period, region, and vaccine, and affected by the perceived benefits, barriers, and sociodemographic factors [7]. It can also be caused by fake news, misunderstanding and conspiracy spread rapidly online via social media [8,9]. Vaccine hesitancy in COVID-19 is an important global issue and barrier to achieve herd immunity. According to a recent global survey, around 40% and 50% of all respondents would not be willing or were unsure to take a COVID-19 vaccine, although there was a wide variation among different countries [10,11]. To safely achieve herd immunity against COVID-19, compulsory vaccination has been suggested in some countries [12] or providing an incentive in order to increase vaccination uptake [13]. The problem of vaccine hesitancy has been identified in Hong Kong. Local studies indicated that the acceptance for COVID-19 vaccines in Hong Kong population has decreased from 44% in the first wave to 35% in the third wave of COVID-19 in Hong Kong [14]. Similarly, a low vaccine acceptance rate (37%) was also identified in another survey within higher proportion of elderly subjects who represent one of the high-risk groups for COVID-19 [15]. Even though a massive government-funded vaccination campaign with choices and sufficient doses of vaccine has been launched for around four months, the uptake rate of the second dose was only about 19.7% of the total population as of 24 June 2021 [16]. The objectives of this study were to examine the uptake and acceptance rate of different COVID-19 vaccines in Hong Kong and investigate the facilitators and barriers of choices and willingness to receive COVID-19 vaccines.

## 2. Materials and Methods

### 2.1. Settings and Subject Recruitment

We performed a population-based, telephone survey in the general population of Hong Kong from 23 April 2021 to 8 May 2021 via random sampling. The survey was started after vaccination has been rolled out for 2 months. Eligible participants were (a). aged over 18 years old; (b). able to understand and speak Cantonese or Mandarin; (c). residents in Hong Kong during the study period; and (d). able to consent participating interview. We targeted adults as subjects aged 18 or above were recommended to receive free COVID-19 vaccine by the government at the time of the study, with a choice between Sinovac (China) [Coronavac] and BioNTech/Fosun (Germany) [Comirnaty]. The present study made reference from the methodology of a telephone survey for the general population on vaccine acceptance conducted in 2020 [15]. The sample population was selected by a Telephone Interview System (TIS) system using simple random sampling where one telephone number was adopted as one randomization unit [17]. We gave at least three telephone attempts when a phone number had not been answered. If the respondent was willing to join the survey but temporarily unavailable, a mutually convenient time was scheduled to administer the survey.

### 2.2. Questionnaires for Telephone Surveys

A survey was devised and face-validated by a panel of microbiologists, epidemiologists, primary care clinicians, nursing specialists, and academics in public health (File S1). The survey was firstly designed by an experienced microbiologist with relevant experience in studies related to COVID-19 vaccine and a healthcare professional with extensive expertise in both clinical and public health research. A meeting involving these interdisciplinary professionals was organized to further refine the instrument. The survey was pilot-tested in a random sample of 50 household residents by telephone, and was critically revised based on the pilot responses. It consisted of survey items in Chinese that collected participants’ attitude and perception of receiving COVID-19 vaccine and policies that could enhance its uptake.

The major domains of the survey include: (1). Socio-demographic date: age, gender, educational level (primary or below vs. secondary vs. tertiary or above), employment status (full-time vs. part-time vs. unemployed vs. retired), changes in income before and during the pandemic, and receipt of public subsidies; (2). Past medical history, including COVID-19 vaccination status, use of chronic medications, and previous history of COVID-19; (3). Perceived susceptibility, perceived severity and perceived benefits of receiving COVID-19 vaccines; (4). Whether COVID-19 vaccination is considered as the most effective strategy to combat the pandemic at present; and (5). Perception on provision of vaccine choice. Survey items (3) to (5) used a Likert scale of 1–4 (“totally disagree” to “totally disagree”). We also enquired: (6). Perceived necessity to implement compulsory vaccination in various high-risk populations; and (7). Perceived effectiveness of vaccine incentives. Among those were not vaccinated and had no intention to be vaccinated: (8). Perceived barriers of receiving COVID-19 vaccines; and (9). Attitude towards the potential enablers of COVID-19 vaccination were explored. Survey items (6) to (10) used a sliding scale of 1–10.

### 2.3. Data Processing and Analysis

The age-sex distribution of the data was further adjusted by the related population distribution among those aged 18 years or older (excluding foreign domestic helpers), provided by the Census and Statistics Department of the Hong Kong government [18]. The primary outcome variable was the proportion expressing intention to be vaccinated among subjects who had not received COVID-19 vaccine. We assumed 50% as the proportion in the outcome, as this generated the maximum sample size. A sample size of approximately 1068 participants could achieve a precision level of less than 0.03, from the formula: “precision = 1.962 × √[(p) × (1 − p)/N]”. All the data were entered into a software spreadsheet and analyzed by the Statistical Package for Social Sciences (SPSS) version 21.0 and AMOS version 23 (Armonk, NY, USA: IBM Corp). Surveys with missing data in more than 10% of the total survey items were excluded from our analysis. For surveys with missing data in less than 10%, we employed multiple imputation in our descriptive analysis. A descriptive analysis of the levels of enabling factors, obstacles and perception of COVID-19 vaccination was performed using Analysis of Variance (ANOVA) and Chi-Square tests for continuous and categorical variables, respectively. We conducted an age-stratified analysis for each outcome variable. All *p* values ≤ 0.05 in the analysis were regarded as statistically significant.

## 3. Results

### 3.1. Participant Characteristics

The response rate was 40%, and seven subjects were excluded because they had COVID-19 before. As a result, 1195 eligible subjects were analyzed. The proportion of subjects aged 18–39 years, 40–59 years and ≥60 years was 30.2%, 38.0% and 31.8%, respectively (Table 1). Among them, 47.8% were male, and 12.6% reported being current recipients of government subsidies. Most of them had full time or part time jobs (58.2%) and attained secondary (38.5%) or tertiary educational levels (47.3%). Approximately 26.6% required use of chronic medications, and 10.4% of them knew COVID-19 patients from their social circle.

### 3.2. Vaccination Status

**Figure 1:**
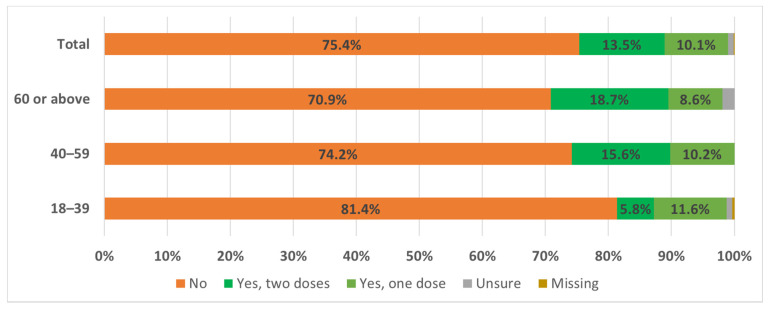
Acceptance rate of COVID-19 vaccine in Hong Kong.

To adjust for the difference in characteristics between participants and the general population in Hong Kong, we applied weighting with the general population in the analysis (Table S1). Majority (75.4%) of respondents had not received any COVID-19 vaccine (Figure 1), whilst only 10.1% and 13.5% had received one and two vaccine doses, respectively. Those who had received COVID-19 vaccine had a roughly equal preference to Sinovac [Coronavac] (43.5%) and BioNTech/Fosun [Comirnaty] (46.9%), with significantly more young subjects aged 18–39 years chosen BioNTech (66.6% vs. 27.2%). All respondents who had received the first dose reported their intention to receive the second dose. Among those who had not received any COVID-19 vaccine, only 25.1% considered vaccination within the subsequent 6 months. This low proportion is irrespective of age groups (22.2%, 29.5% and 23.3% in subjects aged 18–39 years, 40–59 years and ≥60 years, respectively).

### 3.3. Perception towards COVID-19 and Its Vaccine

The majority (73.9%) perceived their risk of contracting COVID-19 as low, with a higher proportion in younger individuals (81.9% vs. 75.4% vs. 64.3% for those aged 18–39 years, 40–59 years and ≥60 years, respectively). Most perceived COVID-19 as a severe disease (69.2%), and the COVID-19 vaccine was effective to reduce disease severity or serious complications (65.9%). In addition, most participants agreed that vaccination was the most effective strategy to combat the COVID-19 pandemic (60.2%) and this proportion was higher in older individuals (65.3% in people aged ≥ 60 years vs. 50.7% in those aged 18–39 years, *P* < 0.001).

### 3.4. Provision of Vaccine Choice

The majority agreed that there should be more than one vaccine for their own choice (79.2%), and that healthcare professionals should recommend the vaccine tailored to the study participants (74.4%). Most did not agree that the government should provide only one vaccine suited for the general population (81.1%), especially among the younger subjects aged 18–39 years (90.9%). However, most considered that there was a lack of comprehensive information on vaccines in the presence of more than one, and this could lead to their hesitancy to receive vaccines (74.7%). Regarding factors influencing their choice of vaccines, their adverse effects (score 7.99 out of 10, 95% C.I. 7.85, 8.13), their efficacy (7.97, 95% C.I. 7.83, 8.12), and the awareness of death cases after vaccination (7.54, 95% C.I. 7.38, 7.70) received the highest scores. The vaccine choice among the younger group was more likely to be influenced by vaccine efficacy (*P* < 0.001), having heard of adverse effects after vaccination (*P* = 0.041), having heard of death cases after vaccination (*P* = 0.021), and country of origin (*P* = 0.002). On the contrary, the vaccine choice score was higher among the older group, who was more likely to be influenced by choices made by government officials (*P* = 0.008)

### 3.5. Perception towards Ways for Increasing Vaccination Rate: Compulsory Vaccination/Cash Incentive

We asked the participants if they agree the proposed five population groups to receive COVID-19 vaccine in a compulsory manner. Around half of the respondents supported the compulsory vaccination among people who provide essential social services such as policemen and firemen (59.3%), followed by foreign domestic helpers (51.1%), personnel working in hospitals and institutions (49.9%), people requiring frequent contact with others (49.6%) and teachers (41.7%). The proportion of agreement for compulsory vaccination was higher in older than younger individuals.

More than 70% of the participants did not agree the government to use cash as an incentive to encourage COVID-19 vaccination in overall and all age groups. Among those who agreed with the use of cash, most (65.2%) suggested from a list of options (HK$100–1000) the highest amounts, which ranged from HK$500 to HK$1000 (US$64 to US$129).

### 3.6. Barriers and Facilitation Measure to Acquire Vaccine among Unvaccinated Subjects

Respondents who had not received vaccine and expressed that they had no intention to receive in the coming six months were directed to questions on the proposed uptake barriers and facilitation measure to acquire vaccination. From a scale of 0–10, the respondents were requested to rate the impact of possible barriers of receiving the vaccine (Table 2). The barriers with the highest score were “having heard of cases with serious adverse events or death after vaccination” (8.17, 95% C.I. 7.99, 8.35), followed by “lack of confidence on governmental recommendations” (7.69, 95% C.I. 7.47, 7.91), “waiting for a better vaccine” (7.29, 95% C.I. 7.07, 7.52), and “lack of confidence on the vaccine manufacturer and its country of origin” (7.11, 95% C.I. 6.89, 7.33). For facilitation measures (Table 3), the highest score was for “vaccine passports for overseas travel” (4.44, 95% C.I. 4.18, 4.71), “granting of leaves on the day of vaccination and the day after” (3.77, 95% C.I. 3.49, 4.05), “allowance to enter entertainment venues” (3.74, 95% C.I. 3.50, 3.99), and “relaxing mandatory isolation” (3.56, 95% C.I. 3.31, 3.80) (Table 3). However, none of these measures reached an average score of 5 out of 10. The score was higher among older group in terms of the facilitation measures of relaxing restrictions on visiting policies in hospitals and healthcare facilities (*P* = 0.014).

For the personal influence of uptake with the vaccine, doctors’ recommendation was rated as the most significant in enhancing COVID-19 vaccine uptake (6.32, 95% C.I. 6.11, 6.54), followed by family members or relatives (3.76, 95% C.I. 3.54, 3.97), colleagues and friends (3.04, 95% C.I. 2.85, 3.23), employers (2.70, 95% C.I. 2.48, 2.92) and the government (2.06, 95% C.I. 1.86, 2.26). The score was higher among older group in terms of the influence by the recommendations from government (*P* = 0.001).

## 4. Discussion

The current study represents the first population-based survey on vaccine hesitancy after the COVID-19 vaccination program was rollout in Hong Kong. At the time of the survey, the acceptance was low at 6.1% [19], and no incentive program for vaccinated residents was initiated. Our findings predicted that only 25% of the unvaccinated population ended up receiving vaccination within the subsequent 6 months. If so, the estimated eventual uptake rate could only reach around 30% within this year, which is consistent to our previous survey conducted prior to the launching of vaccination campaign [15]. There is a concern that the uptake rate of COVID-19 vaccine among Hong Kong citizens remains low even with free-choice of COVID-19 vaccine type, and with sufficient dose to cover its population. The low uptake rate may be explained by the respondents’ low risk perception regarding OVID-19 infection, in a time period corresponding to the end of the fourth pandemic wave in Hong Kong when the survey was conducted. Even though the local residents still had doubts about the vaccination, more than half of the respondents supported making the vaccination compulsory for certain groups of the local population. The suggested compulsory vaccinated groups included those who provide essential social services such as policemen and firemen, people requiring frequent contact with others, healthcare workers, as well as foreign domestic helpers. The finding showed higher agreement on supporting compulsory vaccination when is compared to a Western Asia study which indicated only around one third of healthcare workers supporting compulsory COVID-19 vaccine for all citizens and residents [20]. However, the compulsory COVID-19 vaccination may raise legally enforceable [21] or any stigmatization or discrimination happened afterward [22]. All of these should be explored in further studies for discussion.

On the other hand, most respondents expressed a high level of perceived severity of disease once being infected. Majority of them also acknowledged vaccination was an effective public health measure to control the COVID-19 pandemic and was effective to reduce severity or serious complications even though high vaccine hesitancy among the local population, and this proportion was higher in elder individuals. The major barriers may be some misunderstandings and preconceived notions about the vaccines, including fear of fatal reactions or deaths after vaccination despite the government’s attempt at clarification. The respondents also expressed lack of confidence to the government, vaccine manufacturer and its place of origin. Most people seemed to adopt a “wait and see” approach or to wait for a “better” vaccine which hindered their willingness to become vaccinated [23,24]. The discrepancy between people’s perception and behavior reveals an urgency for policy makers to revamp the present immunization strategies [25]. In addition, the happened fatal side effects after vaccination at the beginning of campaign locally [26] and worldwide [27] may shapely reduce people’s confidence towards the existing COVID-19 vaccination. Instead, recommendation from physicians was considered as the most significant cue to actions, far more important than the recommendation from other parties such as family members or relatives, colleagues and friends, employers, and the government. Given the evidence of safety and efficacy of COVID-19 Vaccines [28], the government should more actively involve physicians in promoting the immunization campaign and to eliminate any public ignorance which is unfavorable to vaccine uptake in local population.

Our previous research study has identified governmental recommendation as an important driver for COVID-19 vaccine uptake [15] before the launching of the government-subsidized vaccination campaign in Hong Kong but “lack of trust in government recommendations” became the second biggest reason for hesitancy according to our findings. A European study also indicated there was an association between political beliefs and attitudes towards vaccine [29]. Therefore, the available information of the vaccination program should be more transparent and comprehensive, to increase the trust and minimize people’s hesitancy to vaccination. We realize it is difficult to modify trust to governmental recommendations within a short period of time. Hence, we suggest other facilitation measures which could improve the vaccination uptake rate. Since the strongest barrier of vaccination was “having heard of cases with serious adverse events or death after vaccination” (having the highest score of 8.17, 95% C.I. 7.99, 8.35), we recommend healthcare professionals in the community could actively offer health educational talks and counselling with emphasis on the safety profile of the vaccine. In addition, our findings highlighted that multiple incentives may be required and a basket of incentive scheme could be formulated by the government. In addition, policy makers, experts and healthcare professionals should join effort to have continuous promotion and education to public about the safety and efficacy of available vaccines in prevention from COVID-19 infection and advise people to be vaccinated as soon as possible instead of waiting for a “better” vaccine.

For vaccine incentive, it was found that a system of vaccine passports for overseas travel would be the most appealing inoculation incentive. Other suggested incentives included granting of leaves on the day of vaccination and the day after, allowance to enter entertainment venues, and relaxing mandatory isolation. However, all of these facilitators only have a low to medium effect on acceptance, in contrast to the strong effects of various barriers. Cash as an incentive has been advocated in the beginning of rollout program. However, most of the participants disagreed with this policy. Overall, no single incentive is strong enough to promote vaccination but multiple facilitators for different groups of population are needed to encourage vaccine uptake.

## 5. Conclusions

COVID-19 remains as a global threat that may not subside in a foreseeable future. Even though the vaccine uptake rate remains low among the local population, we note that the public still perceived COVID-19 as a severe disease, and the majority regard vaccination as the most effective strategy to combat the pandemic. Hence, the government should strengthen public education and information dissemination to tackle vaccine hesitancy. The government should proactively provide transparent and comprehensive information of the vaccination, work with physicians to eliminate any public misconception, and make use of multiple incentives measures. The findings of the current study provide evidence-based support in formulation and implementation of vaccination strategies. However, further studies are needed to confirm the findings as the questionnaire used was only face validated.

## Figures and Tables

**Table 1:** Characteristics of the participants (*n* = 1195).

	Number	Percentage
**Gender**		
Male	570	47.7%
Female	625	52.3%
**Age**		
18–39 years	361	30.2%
40–59 years	453	38.0%
≥60 years	381	31.9%
**Government subsidies**		
Yes	151	12.6%
No	1017	85.1%
Missing	27	2.3%
**Working status**		
Full time/part time	695	58.2%
No	480	40.2%
Missing	20	1.7%
**Education**		
Primary	149	12.5%
Secondary	461	38.6%
Tertiary	564	47.2%
Missing	21	1.8%
**Chronic conditions**		
Yes	318	26.6%
No	862	72.1%
Unsure	4	0.3%
Missing	11	0.9%
**Knew COVID-19 patients from their social circle**		
Yes	122	10.2%
No	1045	87.4%
Unsure	15	1.3%
Missing	13	1.1%

**Table 2:** Score rating on barriers to acquire vaccination among unvaccinated subjects.

Age Group	N	Mean	S.D.	S.E.	95% Confidence Interval		P *
**“Having heard of cases with serious adverse events or death after vaccination”**							0.005
18–39	240	8.39	2.251	0.145	8.10	8.67	
40–59	226	8.36	2.148	0.143	8.08	8.65	
60 or above	209	7.72	2.812	0.195	7.33	8.10	
Total	674	8.17	2.423	0.093	7.99	8.35	
**“Lack of confidence in the vaccine manufacturer and its country of origin”**							0.005
18–39	238	7.54	2.591	0.168	7.21	7.88	
40–59	224	7.05	3.029	0.202	6.65	7.45	
60 or above	198	6.66	2.985	0.212	6.24	7.08	
Total	660	7.11	2.883	0.112	6.89	7.33	
**“Waiting for a better vaccine” as barrier to you to get vaccinated?**							0.269
18–39	241	7.45	2.697	0.174	7.11	7.79	
40–59	219	7.38	2.977	0.201	6.98	7.77	
60 or above	201	7.01	3.236	0.228	6.56	7.46	
Total	660	7.29	2.963	0.115	7.07	7.52	
**“Confusing information about vaccines”**							0.406
18–39	240	6.94	2.838	0.183	6.58	7.30	
40–59	223	6.71	3.022	0.202	6.31	7.11	
60 or above	204	6.56	3.346	0.234	6.09	7.02	
Total	667	6.75	3.062	0.119	6.51	6.98	
**“Lack of confidence in governmental recommendations”**							0.001
18–39	242	8.09	2.611	0.168	7.76	8.42	
40–59	226	7.82	3.003	0.200	7.42	8.21	
60 or above	206	7.10	3.093	0.216	6.67	7.52	
Total	673	7.69	2.922	0.113	7.47	7.91	
**“Lack of confidence in the efficacy of vaccine”**							0.376
18–39	236	5.63	2.567	0.167	5.30	5.96	
40–59	221	5.45	2.907	0.196	5.07	5.84	
60 or above	201	5.25	2.908	0.205	4.85	5.66	
Total	659	5.45	2.790	0.109	5.24	5.67	
**“Inconvenient in getting to vaccination venue”**							0.109
18–39	241	2.35	2.413	0.156	2.04	2.66	
40–59	222	2.81	2.824	0.190	2.44	3.18	
60 or above	198	2.35	2.739	0.195	1.96	2.73	
Total	661	2.50	2.660	0.103	2.30	2.71	
**“Current health condition not suitable” as a barrier to you to get vaccinated?**							<0.001
18–39	237	4.90	3.440	0.223	4.46	5.34	
40–59	219	6.44	3.490	0.236	5.97	6.90	
60 or above	203	6.98	3.062	0.215	6.56	7.41	
Total	659	6.05	3.457	0.135	5.79	6.32	

* *P* values were generated from the Analysis of Variance (ANOVA) comparing the difference in means between age groups; S.D. represents Standard Deviation; S.E. represents Standard Error.

**Table 3:** Score rating on facilitation measures to acquire vaccination among unvaccinated subjects.

Age Group	N	Mean	S.D.	S.E.	95% Confidence Interval		P *
**“Granting reasonable travel expense allowance”**							0.855
18–39	243	2.12	2.477	0.159	1.81	2.43	
40–59	224	2.01	2.788	0.186	1.64	2.38	
60 or above	208	2.15	2.713	0.188	1.77	2.52	
Total	674	2.09	2.653	0.102	1.89	2.29	
**“Granting leaves on the day of vaccination and the day after”**							0.076
18–39	242	4.19	3.505	0.225	3.75	4.64	
40–59	224	3.60	3.789	0.253	3.10	4.10	
60 or above	208	3.46	3.669	0.254	2.96	3.96	
Total	674	3.77	3.661	0.141	3.49	4.05	
**“Vaccine passports for overseas travel”**							0.364
18–39	242	4.61	3.343	0.215	4.18	5.03	
40–59	220	4.52	3.606	0.243	4.04	5.00	
60 or above	202	4.16	3.473	0.244	3.67	4.64	
Total	664	4.44	3.471	0.135	4.18	4.71	
**“Relaxing restrictions on religious activities”**							0.134
18–39	242	2.23	2.674	0.172	1.89	2.57	
40–59	225	2.54	3.279	0.219	2.11	2.98	
60 or above	208	2.82	3.423	0.237	2.35	3.29	
Total	674	2.52	3.129	0.120	2.28	2.75	
**“Relaxing restrictions on visiting policies in hospitals and healthcare facilities”**							0.014
18–39	241	3.21	3.003	0.193	2.83	3.59	
40–59	222	3.20	3.334	0.224	2.76	3.64	
60 or above	209	3.99	3.377	0.234	3.53	4.46	
Total	671	3.45	3.249	0.125	3.21	3.70	
**“Relaxing mandatory isolation”**							0.241
18–39	237	3.38	3.022	0.196	2.99	3.76	
40–59	222	3.85	3.395	0.228	3.40	4.30	
60 or above	205	3.45	3.180	0.222	3.01	3.89	
Total	664	3.56	3.201	0.124	3.31	3.80	
**“Resumption of face–to–face teaching in schools”**							0.159
18–39	240	3.21	3.270	0.211	2.79	3.62	
40–59	221	3.72	3.646	0.245	3.24	4.21	
60 or above	201	3.15	3.383	0.239	2.68	3.62	
Total	661	3.36	3.438	0.134	3.10	3.62	
**“Allowance to enter entertainment venues”**							0.643
18–39	242	3.79	3.169	0.204	3.39	4.19	
40–59	222	3.85	3.427	0.230	3.40	4.31	
60 or above	208	3.57	3.207	0.223	3.13	4.01	
Total	671	3.74	3.265	0.126	3.50	3.99	
**“Recommendation by doctors”**							0.299
18–39	243	6.50	2.424	0.156	6.20	6.81	
40–59	225	6.35	3.042	0.203	5.95	6.75	
60 or above	214	6.09	3.157	0.216	5.66	6.51	
Total	681	6.32	2.877	0.110	6.11	6.54	
**“Recommendation by family members or relatives”**							0.182
18–39	242	3.49	2.600	0.167	3.16	3.82	
40–59	223	3.86	2.935	0.197	3.47	4.24	
60 or above	209	3.96	2.983	0.206	3.55	4.37	
Total	674	3.76	2.838	0.109	3.54	3.97	
**“Recommendation by colleagues and friends”**							0.333
18–39	241	3.18	2.454	0.158	2.87	3.49	
40–59	226	2.84	2.659	0.177	2.49	3.19	
60 or above	212	3.08	2.493	0.171	2.74	3.42	
Total	678	3.04	2.536	0.097	2.85	3.23	
**“Recommendation by employers”**							0.646
18–39	238	2.83	2.670	0.173	2.49	3.17	
40–59	221	2.59	2.990	0.201	2.19	2.98	
60 or above	198	2.67	3.034	0.216	2.24	3.10	
Total	656	2.70	2.890	0.113	2.48	2.92	
**“Recommendation by the government”**							0.001
18–39	241	1.79	2.412	0.155	1.48	2.10	
40–59	221	1.80	2.516	0.169	1.46	2.13	
60 or above	207	2.64	2.871	0.200	2.25	3.04	
Total	668	2.06	2.621	0.101	1.86	2.26	

* *P* values were generated from the Analysis of Variance (ANOVA) comparing the difference in means between age groups; S.D. represents Standard Deviation; S.E. represents Standard Error.

## Data Availability

The data presented in this study are available on request from the corresponding author.

## Supplementary Materials

The following are available online at https://www.mdpi.com/article/10.3390/vaccines9111250/s1, File S1: Survey on Views about COVID-19 Vaccination, Table S1: Status and Perceptions towards COVID-19 Vaccine.
