# Supplementary material for: COVID-19 Vaccine Hesitancy in a City with Free Choice and Sufficient Doses
Source: Vaccines (Basel). 2021 Oct 28;9(11):1250. doi: 10.3390/vaccines9111250 (PMC8618889; doi:10.3390/vaccines9111250)
Supplement: Supplementary file 1 [file vaccines-09-01250-s001.zip › vaccines-1387430-supplementary.pdf]

File S1. **Survey on Views about COVID-19 Vaccination**

Hello, we are calling from Hong Kong Institute of Asia-Pacific Studies, CUHK. We are conducting a survey on views about COVID-19 vaccination. All information will be kept strictly confidential. This survey takes about ten minutes and it is taken voluntarily. You can withdraw from this survey anytime. If you have any enquiries, please contact us at 39436741. Do you agree to take the survey?

【Commission: The Jockey Club School of Public Health and Primary Care & Department of Microbiology, CUHK (Enquiry: Mr Chu 22528760)】

【If the respondent has done this survey, end the interview.】

【Landline survey】

L_SEL To prevent bias at a certain groups of people, we need to perform random sampling. Except for domestic helpers, how many family members, including yourself, aged 18 or above live in this flat?

____ people【If there is more than one person, select the person who has his/her birthday next from all those present. If the selected person is not at home, please make an appointment to call again.】

【Mobile phone survey】

M_SEL The target population of this survey is Hong Kong residents aged 18 or above. Do you belong to this group?

【If the respondent is not a Hong Kong resident, end the interview.】

【Survey starts】

GENDER

【Fill in by the interviewer. If it cannot be distinguished by voice, ask "What is your gender?"】

1. Male
2. Female
7. Others
9. Refuse to answer

AGE What age group do you belong to? Aged 18 - 29, 30 – 39, 40 – 49, 50 – 59, 60 - 69，or aged 70 or above?

1. Aged 18 - 29
2. Aged 30 - 39
9. Refuse to answer

3. Aged 40 - 49
4. Aged 50 - 59
5. Aged 60 - 69
6. Aged 70 or above

Q1 How do you perceive your risk of being infected with COVID-19 in the coming 12 months? Very high, high, low or very low?

1. Very high
2. High
3. Low
4. Very low

8. Don’t know/Uncertain
9. Refuse to answer

Q2 If you are infected with COVID-19, how serious do you think it will affect your health? Very serious, serious, not serious or not serious at all?

1. Very serious
2. Serious
3. Not serious
4. Not serious at all

8. Don’t know/Uncertain
9. Refuse to answer

Q3 How do you perceive the COVID-19 vaccine is effective to reduce disease severity and serious complications? Definitely, probably, probably not or definitely not?

1. Definitely
2. Probably
3. Probably not
4. Definitely not

8. Don’t know/Uncertain
9. Refuse to answer

The following are some statements about the COVID-19 vaccination. How much do you agree with these statements? You can choose “strongly agree”, “agree”, “disagree” or “strongly disagree”.

Q4 How much do you agree “vaccination is the most effective way to combat the COVID-19 pandemic”?

1. Strongly agree
2. Agree
3. Disagree
4. Strongly disagree

8. Don’t know/Uncertain
9. Refuse to answer

Q5 How much do you agree “if more different types of vaccines are available, it allows me to choose the most suitable vaccine”?

1. Strongly agree
2. Agree
3. Disagree

8. Don’t know/Uncertain
9. Refuse to answer

4. Strongly disagree

Q6 How much do you agree “healthcare professionals should recommend the vaccine tailored to my health condition”?

1. Strongly agree
2. Agree
3. Disagree
4. Strongly disagree
8. Don’t know/Uncertain
9. Refuse to answer

Q7 How much do you agree “the lack of comprehensive information on vaccines in the presence of more than one choice will lead to hesitancy to receive vaccines”?

1. Strongly agree
2. Agree
3. Disagree
4. Strongly disagree
8. Don’t know/Uncertain
9. Refuse to answer

Q8 How much do you agree “the government should only provide one best vaccine to its citizens”?

1. Strongly agree
2. Agree
3. Disagree
4. Strongly disagree
8. Don’t know/Uncertain
9. Refuse to answer

R1 If their health condition allows, do you support mandatory vaccination should be applied for the following categories of people? You can choose “strongly support”, “support”, “oppose” or “strongly oppose”.

Q9 How much do you support for “personnel working in hospitals and residential care homes”?

1. Strongly support
2. Support
3. Oppose
4. Strongly oppose
8. Don’t know/Uncertain
9. Refuse to answer

Q10 How much do you support for “personnel working in service industry who require frequent contact with customers”?

1. Strongly support
8. Don’t know/Uncertain

2. Support
3. Oppose
4. Strongly oppose
9. Refuse to answer

Q11 How much do you support for “personnel who provide public emergency services, e.g. police, firefighters”?

1. Strongly support
2. Support
3. Oppose
4. Strongly oppose
8. Don’t know/Uncertain
9. Refuse to answer

Q12 How much do you support for “foreign domestic helpers”?

1. Strongly support
2. Support
3. Oppose
4. Strongly oppose
8. Don’t know/Uncertain
9. Refuse to answer

Q13 How much do you support for “teachers”?

1. Strongly support
2. Support
3. Oppose
4. Strongly oppose
8. Don’t know/Uncertain
9. Refuse to answer

Q14 How much do you support the government should use cash as an incentive to encourage COVID-19 vaccination?

1. Strongly support【To Q15】
2. Support【To Q15】
3. Oppose【To Q16】
4. Strongly oppose【To Q16】
8. Don’t know/Uncertain【To Q16】
9. Refuse to answer【To Q16】

【Available for respondents who choose option 1/2 in Q14】
Q15 How much cash incentive is appropriate? $100, $300, $500, $700 or $1,000?

1. $100
2. $300
3. $500
4. $700
5. $1,000
7. Others (please specify)
8. Don’t know/Uncertain
9. Refuse to answer

Q16 Have your received COVID-19 vaccine? (1) Never, (2) Completed two doses, (3) Received the first dose and will take the second dose or (4) Received the first dose but will not take the second dose

1. Never (including people who will book the vaccination)【To Q17】
2. Completed two doses【To Q39a】
3. Received the first dose and will take the second dose【To Q39a】
4. Received the first dose but will not take the second dose【To R2】
8. Don't know/Uncertain【To Q17】
9. Refuse to answer【To Q39b】

【Available for respondents who choose option 1 in Q16】

Q17 Are you planning to get the COVID-19 vaccine or make an appointment for vaccination in the future?

1. Yes/Have made an appointment【To Q39a】 8. Don't know/Uncertain【To R2】
2. No【To R2】 9. Refuse to answer【To Q39b】

【Available for respondents who choose option 4 in Q16 or option 2/8 in Q17】

R2 The following are some reasons for not getting the COVID-19 vaccine (or not getting the second dose of vaccine). Score 0 represents "no effect", score 5 represents "medium effect", and score 10 represents "the greatest effect", how much do the following reasons affect your willingness in vaccine uptake?

Q18 From 0 to 10, how do you score "having heard of cases with serious adverse events or death after vaccination" as barrier to you to get vaccinated?

__________【0-10: the higher the score, the greater the effect of the cause】
88. Don't know/Uncertain
99. Refuse to answer

Q19 From 0 to 10, how do you score "lack of confidence in the vaccine manufacturer and its country of origin" as barrier to you to get vaccinated?

__________【0-10: the higher the score, the greater the effect of the cause】
88. Don't know/Uncertain
99. Refuse to answer

Q20 From 0 to 10, how do you score "waiting for a better vaccine" as barrier to you to get vaccinated?

__________【0-10: the higher the score, the greater the effect of the cause】
88. Don’t know/Uncertain
99. Refuse to answer

Q21 From 0 to 10, how do you score "confusing information about vaccines" as barrier to you to get vaccinated?

__________【0-10: the higher the score, the greater the effect of the cause】
88. Don’t know/Uncertain
99. Refuse to answer

Q22 From 0 to 10, how do you score "lack of confidence in recommendations from HKSAR Government" as a barrier to you to get vaccinated?

__________【0-10: the higher the score, the greater the effect of the cause】
88. Don’t know/Uncertain
99. Refuse to answer

Q23 From 0 to 10, how do you score "lack of confidence in the efficacy of vaccine" as a barrier to you to get vaccinated?

__________【0-10: the higher the score, the greater the effect of the cause】
88. Don’t know/Uncertain
99. Refuse to answer

Q24 From 0 to 10, how do you score "inconvenient to go to vaccination venue" as a barrier to you to get vaccinated?

__________【0-10: the higher the score, the greater the effect of the cause】
88. Don’t know/Uncertain
99. Refuse to answer

Q25 From 0 to 10, how do you score "current health condition not suitable" as a barrier to you to get vaccinated?

__________【0-10: the higher the score, the greater the effect of the cause】
88. Don’t know/Uncertain
99. Refuse to answer

R3 The following are some strategies to encourage vaccine uptake among citizens. Score 0 represents "no effect", score 5 represents "medium effect", and

score 10 represents "the greatest effect", how much do the following strategies motivate you to get the COVID-19 vaccine?

Q26 From 0 to 10, how do you score "granting reasonable travel allowances" as a facilitator to you to get vaccinated?

__________【0-10: the higher the score, the greater the effect of the cause】
88. Don't know/Uncertain
99. Refuse to answer

Q27 From 0 to 10, how do you score "granting leaves on the day of vaccination and the day after" as a facilitator to you to get vaccinated?

__________【0-10: the higher the score, the greater the effect of the cause】
88. Don't know/Uncertain
99. Refuse to answer

Q28 From 0 to 10, how do you score "vaccine passports for overseas travel" as a facilitator to you to get vaccinated?

__________【0-10: the higher the score, the greater the effect of the cause】
88. Don't know/Uncertain
99. Refuse to answer

R4 It is assumed that "immunity passport" is implemented in Hong Kong to facilitate citizens in participation of certain activities or access to certain venues. Score 0 represents "no effect", score 5 represents "medium effect", and score 10 represents "the greatest effect", how much do following relaxation of social distancing measures motivate you to get the COVID-19 vaccine?

Q29 From 0 to 10, how do you score "relaxing restrictions on religious activities" as a facilitator to you to get vaccinated?

__________【0-10: the higher the score, the greater the effect of the cause】
88. Don't know/Uncertain
99. Refuse to answer

Q30 From 0 to 10, how do you score "relaxing restrictions on visiting policies in hospitals and healthcare facilities" as a facilitator to you to get vaccinated?

__________【0-10: the higher the score, the greater the effect of the cause】

88. Don’t know/Uncertain
99. Refuse to answer

Q31 From 0 to 10, how do you score "relaxing mandatory quarantine" as a facilitator to you to get vaccinated?

__________【0-10: the higher the score, the greater the effect of the cause】
88. Don’t know/Uncertain
99. Refuse to answer

Q32 From 0 to 10, how do you score "resumption of face-to-face teaching in schools" as a facilitator to you to get vaccinated?

__________【0-10: the higher the score, the greater the effect of the cause】
88. Don’t know/Uncertain
99. Refuse to answer

Q33 From 0 to 10, how do you score "allowing to enter entertainment venues, such as restaurants, fitness rooms, sports fields, swimming pools, bars and karaoke box, etc." as a facilitator to you to get vaccinated?

__________【0-10: the higher the score, the greater the effect of the cause】
88. Don’t know/Uncertain
99. Refuse to answer

R5 The following are the advices from different parties. Score 0 represents "no effect", score 5 represents "medium effect", and score 10 represents "the greatest effect", how much do the advice of the following parties as a facilitator to you to get vaccinated?

Q34 From 0 to 10, how do you score "recommendation by doctors" as a facilitator to you to get vaccinated?

__________【0-10: the higher the score, the greater the effect of the cause】
88. Don’t know/Uncertain
99. Refuse to answer

Q35 From 0 to 10, how do you score "recommendation by family members or relatives" as a facilitator to you to get vaccinated?

__________【0-10: the higher the score, the greater the effect of the cause】
88. Don’t know/Uncertain

99. Refuse to answer

Q36 From 0 to 10, how do you score "recommendation by colleagues and friends" as a facilitator to you to get vaccinated?

__________【0-10: the higher the score, the greater the effect of the cause】
88. Don’t know/Uncertain
99. Refuse to answer

Q37 From 0 to 10, how do you score "recommendation by employers" as a facilitator to you to get vaccinated?

__________【0-10: the higher the score, the greater the effect of the cause】
88. Don’t know/Uncertain
99. Refuse to answer

Q38 From 0 to 10, how do you score "recommendation by the government" as a facilitator to you to get vaccinated?

__________【0-10: the higher the score, the greater the effect of the cause】
88. Don’t know/Uncertain
99. Refuse to answer

【Available for respondents who have received one or two doses, or have booked the vaccination (choose option 2/3/4 in Q16)】

Q39a Which COVID-19 vaccines have you received (or plan to receive in the future)? Sinovac or BioNTech?

1. Sinovac
2. BioNTech
8. Not yet decided
9. Refuse to answer

【Available for respondents who have not vaccinated (choose option 1/9 in Q16 or choose option 2/8 in Q17)】

Q39b If you have to get your vaccine, which COVID-19 vaccine will you choose? Sinovac or BioNTech?
1. Sinovac
2. BioNTech
3. Wait for a better vaccine
8. Not yet decided/Don’t know/Uncertain
9. Refuse to answer

Q40 Do you know the difference between the two COVID-19 vaccines (Sinovac and BioNTech)? Fully understand, understand, do not understand or not understand at all?

1. Fully understand
2. Understand
3. Do not understand
4. Not understand at all
8. Don’t know/Uncertain
9. Refuse to answer

R5 Score 0 represents "no effect", score 5 represents "medium effect", and score 10 represents "the greatest effect", how much do the following factors affect your preference in choosing COVID-19 vaccine?

Q41 From 0 to 10, how do you score "vaccine efficacy" as a factor influencing your choice of vaccine?

__________【0-10: the higher the score, the greater the effect of the cause】
88. Don’t know/Uncertain
99. Refuse to answer

Q42 From 0 to 10, how do you score "adverse effects after vaccination" as a factor influencing your choice of vaccine?

__________【0-10: the higher the score, the greater the effect of the cause】
88. Don’t know/Uncertain
99. Refuse to answer

Q43 From 0 to 10, how do you score "death cases after vaccination" as a factor influencing your choice of vaccine?

__________【0-10: the higher the score, the greater the effect of the cause】
88. Don’t know/Uncertain
99. Refuse to answer

Q44 From 0 to 10, how do you score "vaccine manufacturing technology (e.g. traditional or new approach)" as a factor influencing your choice of vaccine?

__________【0-10: the higher the score, the greater the effect of the cause】
88. Don’t know/Uncertain
99. Refuse to answer

Q45 From 0 to 10, how do you score "country of origin" as a factor influencing your choice of vaccine?

__________【0-10: the higher the score, the greater the effect of the cause】
88. Don't know/Uncertain
99. Refuse to answer

Q46 From 0 to 10, how do you score "convenience in overseas travels (e.g. being accepted by foreign countries)" as a factor influencing your choice of vaccine?

__________【0-10: the higher the score, the greater the effect of the cause】
88. Don't know/Uncertain
99. Refuse to answer

Q47 From 0 to 10, how do you score "choices made by experts" as a factor influencing your choice of vaccine? 【e.g. the vaccine received by experts】

__________【0-10: the higher the score, the greater the effect of the cause】
88. Don't know/Uncertain
99. Refuse to answer

Q48 From 0 to 10, how do you score "choices made by government officials" as a factor influencing your choice of vaccine?【e.g. the vaccine received by government officials】

__________【0-10: the higher the score, the greater the effect of the cause】
88. Don't know/Uncertain
99. Refuse to answer

Q49 From 0 to 10, how do you score "choices made by healthcare workers" as a factor influencing your choice of vaccine?

__________【0-10: the higher the score, the greater the effect of the cause】
88. Don't know/Uncertain
99. Refuse to answer

Q50 From 0 to 10, how do you score "choices made by family, friends, and colleagues" as a factor influencing your choice of vaccine?

__________ 【0-10: the higher the score, the greater the effect of the cause】
88. Don’t know/Uncertain
99. Refuse to answer

Q51 From 0 to 10, how do you score "advice from key pinion leaders (KOL) on the internet" as a factor influencing your choice of vaccine?

__________ 【0-10: the higher the score, the greater the effect of the cause】
88. Don’t know/Uncertain
99. Refuse to answer

Q52 Do you require use of chronic medications?

1. Yes
2. No
8. Don’t know/Uncertain
9. Refuse to answer

Q53 Do you know any person in your social circle diagnosed with COVID-19?

1. Yes
2. No
8. Don’t know/Uncertain
9. Refuse to answer

Q54 Have you been diagnosed with COVID-19?

1. Yes
2. No
8. Don’t know/Uncertain
9. Refuse to answer

EDU
What is your highest level of education? Primary or below, secondary, tertiary level or above

1. Primary or below
2. Secondary (Form 1 -7/matriculation)
9. Refuse to answer

3. Tertiary level or above (diploma, higher diploma, associate degree, bachelor's degree, master's degree, doctoral degree)

WORK

What is your employment status?

1. Employed (full-time/part-time)【To INDUST1】
2. Not in employment【To SUBSIDE】
9. Refuse to answer【To SUBSIDE】

【Available for respondents who are employed】

INDUST1 What field are you in?【Don’t read the field】

1. Manufacturing (e.g. food, clothing, printing, plastic, metal, furniture, chemical manufacturing, etc.)
2. Electricity, gas supply, waste recycling
3. Construction (construction, renovation, civil engineering, etc.)
4. Import/export, wholesale and retail trades
5. Transportation, storage, postal and courier services (driver, air transportation, warehouse, logistics, etc.)
6. Accommodation and food services (hotels, catering, etc.)
7. Publishing, film, recording, programming and broadcasting
8. Information and communications (Telecommunications, IT, Internet, etc.)
9. Financial and insurance (banking, investments, retirement funds, etc.)
10. Real estate (real estate trading, property management, etc.)
11. Professional, scientific and technical services (law, accounting, public relations, surveying, scientific research, design, etc.)
12. Administration and support services (rental and leasing, employment, tourism, security, office administration)
13. Public administration (civil servant, assistants of Legislative Council/District Councils Members)
14. Education (schools, teaching, tutorial schools)
15. Healthcare (hospitals, nursing) (e.g. doctors, nurses, first-aiders)
16. Social welfare and social work services (e.g. non-profit organizations or charitable organizations)
17. Arts, entertainment, recreation or other cultural activities (art, library, sports, societies, religion, etc.)
18. Maintenance and other personal services (repair of motor vehicles, home maintenance, laundry, beauty, private tutoring)

77. Others【please specify】

99. Refuse to answer

【Available for respondents who are employed】

INDUST2 Do you encounter 50 or more people in workplace every day?

1. Yes
2. No
8. Don't know/Uncertain
9. Refuse to answer

【Available for respondents who are employed】

INCOME Comparison of before and after COVID-19, how does the pandemic affect your current monthly income? No impact, increase or decrease?

【If there is a decrease, ask follow-up question: what is the percentage of decrease? 10%, 10% to less than 30%, 30% to less than 50%, 50% or above】

1. No impact
2. Increase
3. Decrease: less than10%
4. Decrease: 10% to less than 30%
5. Decrease: 30% to less than 50%
6. Decrease: 50% or above
8. Don't know/Uncertain
9. Refuse to answer

SUBSIDE Do you receive any allowance (e.g. Comprehensive Social Security Assistance Scheme, Disability Allowance, Old Age Allowance, etc.) from the HKSAR Government?

1. Yes
2. No
9. Refuse to answer

** This is the end of the survey. Thank you for your time, goodbye! **

Table S1. Status and perceptions towards COVID-19 vaccine

	Age Groups (years)								
	**18-39**		**40-59**		**60 or above**		**Total**		
	**Number**	**Percentage**	**Number**	**Percentage**	**Number**	**Percentage**	**Number**	**Percentage**	***P****
**Have you received COVID-19 vaccine?**									<0.001
No	308	81.4%	321	74.2%	272	70.9%	901	**75.4%**	
Yes, two doses	22	5.8%	68	15.6%	71	18.7%	161	**13.5%**	
Yes, one dose	44	11.6%	44	10.2%	33	8.6%	121	**10.1%**	
Unsure	4	0.9%	0	0.0%	7	1.9%	11	0.9%	
Missing	1	0.3%	0	0.0%	0	0.0%	1	0.1%	
**Do you intent to receive the vaccine within the coming 6 months?**									<0.001
Yes	69	**22.2%**	95	**29.5%**	65	**23.3%**	229	**25.1%**	
No	187	59.9%	149	46.5%	121	43.6%	458	50.2%	
Unsure	56	17.9%	77	24.0%	92	33.1%	225	24.7%	
Missing	0	0.0%	0	0.0%	0	0.0%	0	0.0%	
**Which vaccine did you choose (Sinovac or BioNTech)?**									<0.001
Sinovac	37	27.2%	94	45.4%	92	54.1%	222	**43.5%**	
BioNTech	90	66.6%	90	43.7%	59	35.1%	240	**46.9%**	
Unsure	7	5.5%	17	8.2%	11	6.8%	36	7.0%	
Missing	1	0.7%	6	2.7%	7	4.0%	13	2.6%	
**How do you perceive your risk of contracting COVID-19 in the coming 12 months?**									<0.001
High	48	12.6%	64	14.9%	61	16.0%	173	14.5%	
Low	311	**81.9%**	327	**75.4%**	246	**64.3%**	884	**73.9%**	

Unsure	21	5.5%	42	9.7%	75	19.7%	138	11.6%	
Missing	0	0.0%	0	0.0%	0	0.0%	0	0.0%	
**Do you perceive COVID-19 as a severe disease?**									<0.001
Yes	257	67.8%	300	69.3%	270	70.5%	827	**69.2%**	
No	105	27.8%	94	21.7%	62	16.2%	262	21.9%	
Unsure	17	4.4%	39	9.0%	51	13.2%	106	8.9%	
Missing	0	0.0%	0	0.0%	0	0.0%	0	0.0%	
**Do you perceive the COVID-19 vaccine is effective to reduce disease severity or serious complications?**									<0.001
Yes	239	62.9%	301	69.8%	247	64.4%	787	**65.9%**	
No	117	30.8%	85	19.7%	70	18.3%	272	22.7%	
Unsure	24	6.3%	46	10.5%	66	17.4%	136	11.4%	
Missing	0	0.0%	0	0.0%	0	0.0%	0	0.0%	
**Do you agree "vaccination is the most effective strategy to combat the COVID-19 pandemic"?**									<0.001
Yes	192	**50.7%**	277	63.9%	250	**65.3%**	719	**60.2%**	
No	178	47.0%	138	31.9%	99	25.8%	415	34.7%	
Unsure	9	2.3%	17	4.0%	32	8.4%	58	4.8%	
Missing	0	0.0%	1	0.2%	2	0.6%	3	0.3%	
**Do you agree "there should be more than one vaccine for you to choose from"?**									0.114
Yes	295	77.7%	342	78.9%	311	81.1%	947	**79.2%**	
No	75	19.8%	73	16.9%	52	13.7%	201	16.8%	
Unsure	9	2.5%	18	4.2%	20	5.2%	47	4.0%	

Missing	0	0.0%	0	0.0%	0	0.0%	0	0.0%	
**Do you agree "healthcare professionals should recommend the vaccine tailored to your health condition"?**									0.066
Yes	290	76.4%	314	72.6%	285	74.4%	889	**74.4%**	
No	72	19.0%	98	22.6%	66	17.3%	236	19.8%	
Unsure	17	4.6%	21	4.8%	32	8.3%	70	5.9%	
Missing	0	0.0%	0	0.0%	0	0.0%	0	0.0%	
**Do you agree "the lack of comprehensive information on vaccines in the presence of more than one choice will lead to hesitancy to receive vaccines"?**									0.025
Yes	299	79.0%	322	74.4%	271	70.8%	892	**74.7%**	
No	71	18.7%	95	22.0%	87	22.8%	254	21.2%	
Unsure	9	2.3%	16	3.6%	25	6.5%	49	4.1%	
Missing	0	0.0%	0	0.0%	0	0.0%	0	0.0%	
**Do you agree "the government should only provide one best vaccine to its citizens"?**									<0.001
Yes	28	7.5%	59	13.6%	92	24.0%	179	15.0%	
No	345	**90.9%**	364	84.0%	261	68.2%	970	**81.1%**	
Unsure	6	1.6%	9	2.2%	28	7.3%	43	3.7%	
Missing	0	0.0%	1	0.2%	2	0.5%	3	0.3%	
**Do you support compulsory vaccination for personnel working in hospitals and institutions if their health condition allows?**									<0.001
Yes	156	41.3%	227	52.5%	213	55.6%	597	**49.9%**	
No	209	55.3%	189	43.7%	147	38.4%	546	45.7%	

Unsure	13	3.5%	16	3.8%	23	6.0%	52	4.4%	
Missing	0	0.0%	0	0.0%	0	0.0%	0	0.0%	
**Do you support compulsory vaccination for people requiring frequent contact with others if their health condition allows?**									<0.001
Yes	142	37.5%	218	50.4%	232	60.6%	592	**49.6%**	
No	223	58.9%	204	47.0%	133	34.7%	560	46.8%	
Unsure	14	3.6%	12	2.7%	18	4.7%	43	3.6%	
Missing	0	0.0%	0	0.0%	0	0.0%	0	0.0%	
**Do you support compulsory vaccination for essential service providers, such as policemen and firemen if their health condition allows?**									<0.001
Yes	195	51.5%	247	57.0%	267	69.8%	709	**59.3%**	
No	177	46.8%	167	38.6%	93	24.4%	438	36.6%	
Unsure	6	1.7%	19	4.5%	22	5.8%	48	4.0%	
Missing	0	0.0%	0	0.0%	0	0.0%	0	0.0%	
**Do you support compulsory vaccination for foreign domestic helpers if their health condition allows?**									<0.001
Yes	156	41.2%	225	51.9%	230	59.9%	611	**51.1%**	
No	216	57.1%	190	43.9%	125	32.6%	531	44.5%	
Unsure	7	1.7%	18	4.2%	29	7.5%	53	4.5%	
Missing	0	0.0%	0	0.0%	0	0.0%	0	0.0%	
**Do you support compulsory vaccination for teachers if their health condition allows?**									<0.001
Yes	112	29.5%	189	43.6%	198	51.6%	498	**41.7%**	
No	257	67.9%	221	51.1%	158	41.2%	637	53.3%	

Unsure	10	2.6%	23	5.3%	28	7.2%	60	5.0%	
Missing	0	0.0%	0	0.0%	0	0.0%	0	0.0%	
**Do you agree the government should use cash as an incentive to encourage COVID-19 vaccination?**									<0.001
Yes	102	26.9%	105	24.2%	112	29.3%	319	26.7%	
No	272	71.8%	323	74.7%	242	63.1%	837	70.1%	
Unsure	4	1.0%	5	1.1%	29	7.6%	38	3.2%	
Missing	1	0.2%	0	0.0%	0	0.0%	1	0.1%	
**How much cash incentive do you think is appropriate (for those who respond "Yes" to previous question)?**									0.003
$100	9	8.6%	11	10.8%	11	9.8%	31	9.8%	
$300	11	10.9%	10	9.4%	4	3.8%	25	7.9%	
$500	20	19.4%	23	21.8%	19	16.6%	61	19.2%	
$700	10	9.3%	4	3.7%	11	9.6%	24	7.6%	
$1,000	46	44.7%	42	40.3%	35	30.8%	123	38.4%	
Others	3	2.9%	7	6.6%	10	9.1%	20	6.3%	
Unsure	4	4.1%	8	7.5%	23	20.4%	35	11.0%	
Missing	0	0.0%	0	0.0%	0	0.0%	0	0.0%	

*P values were generated from the Chi-Square tests comparing the difference in proportions between age groups.
